# Intellectual functioning in alpha‐mannosidosis

**DOI:** 10.1002/jmd2.12073

**Published:** 2019-09-21

**Authors:** Sara S. Cathey, Sara M. Sarasua, Richard Simensen, Katie Pietris, Gordon Kimbrell, David Sillence, Callum Wilson, Lucia Horowitz

**Affiliations:** ^1^ Greenwood Genetic Center Greenwood South Carolina; ^2^ Clemson University School of Nursing Clemson South Carolina; ^3^ InSite Solutions Washington District of Columbia; ^4^ Private Practice Mt Pleasant South Carolina; ^5^ Genetic Medicine, Westmead Hospital, Westmead and Genomic Medicine, Faculty of Medicine and Health University of Sydney New South Wales Australia; ^6^ Starship Children's Hospital Auckland New Zealand

**Keywords:** alpha‐mannosidosis, glycoproteinoses, intellectual disability, IQ

## Abstract

Alpha‐mannosidosis is a rare inherited metabolic disorder (OMIM #248500) caused by mutations in the enzyme α‐mannosidase encoded by the gene *MAN2B1*. Patients have distinct physical and developmental features, but only limited information regarding standardized cognitive functioning of patients has been published. Here we contribute intellectual ability scores (IQ) on 12 patients with alpha‐mannosidosis (ages 8‐59 years, 10 males, 2 females). In addition, a pooled analysis was performed with data collected from this investigation and 31 cases obtained from the literature, allowing a comprehensive analysis of intellectual functioning in this rare disease. The initial and pooled analyses show that patients with alpha‐mannosidosis have variable degrees of intellectual disability but show decline in IQ with age, particularly during the first decade of life. Patients treated with hematopoietic stem cell transplantation tend to show stabilized cognitive abilities.

**Synopsis**

Alpha‐mannosidosis patients show rapid decline in cognitive function during the first two decades of life.

## 1 INTRODUCTION

Alpha‐Mannosidosis (OMIM #248500) is caused by a gene defect in the enzyme α‐mannosidase encoded by the gene *MAN2B1*. The disorder is inherited in an autosomal recessive manner. The condition is characterized by developmental delay and intellectual disability, coarse facies, hearing impairment, mild dysostosis multiplex, and immune deficiency. There is a spectrum of severity but three types were described in the older literature prior to genotyping. Those labeled with type 3 had the most severe course with earliest onset and shortest survival. We now know there is no clear genotype‐phenotype correlation.^1^, ^2^, ^3^, ^4^ All of our patients were symptomatic at diagnosis and within the first 2 years of life with concerns for hearing loss, developmental delays or regression, and frequent infections.

Far fewer detailed clinical reports have been published on patients with alpha‐mannosidosis as compared to other groups of lysosomal diseases with approved therapies such as Pompe, Gaucher, Fabry, and the mucopolysaccharidosis (MPS) disorders. At the time of this writing, no approved treatment is on the market in the United States of America. However, hematopoietic stem cell transplant (HCT) has been shown to positively impact developmental outcome in alpha‐mannosidosis in several patients.^5^, ^6^ More recently, enzyme replacement therapy has been approved for treating the non‐neuropathic features of the disease in the EU with orphan drug designation.^2^, ^7^, ^8^

Reports on intellectual function in alpha‐mannosidosis are scarce. One set of measures of effectiveness of therapies is impact on IQ. In this study, we measured IQ in 12 patients with alpha‐mannosidosis to assess severity and changes with age.

## 2 MATERIALS AND METHODS

### 2.1 Participants

Twelve patients with alpha‐mannosidosis were evaluated by licensed and/or certified clinical or developmental psychologists. One patient had previously been treated with cord blood transplantation. Evaluations took place at the Greenwood Genetic Center, South Carolina, in 2009 and 2012 and in New Zealand and Australia in 2009.^9^ Patients were enrolled in the study “Longitudinal Studies of the Glycoproteinoses,” with IRB approved protocol and consent. This data is from a cross‐sectional component of the larger study.

### 2.2 Assessment

#### 2.2.1 Cognitive functioning

Patients were assessed with either the *Kaufman‐Brief Intelligence Test*––*2nd Edition (K‐BIT‐2)*,^10^ the *Developmental Assessment of Young Children (DAYC)*,^11^ or the *Peabody Picture Vocabulary Test*––*4th Edition* 12 depending on the patient's age, severity of intellectual impairment, and fine motor abilities. Scores on the K‐BIT‐2, DAYC, and PPVT‐4 are presented as standard scores (mean = 100, SD = 15). Values below 70 indicate cognitive impairment.

### 2.3 Pooled data from the literature on cognitive function

To better understand the changes in cognitive function with age in a larger sample size of this rare disease, the literature was reviewed to identify case reports for a pooled analysis. Literature‐based data were only included in the pooled analysis if the age at measurement was provided and the IQ was quantified rather than estimated. Fifteen publications with cognitive data on 31 patients were identified.^5^, ^6^, ^13^, ^14^, ^15^, ^16^, ^17^, ^18^, ^19^, ^20^, ^21^, ^22^, ^23^, ^24^, ^25^ The data were analyzed separately for those who had and had not undergone HCT. The Table S2 includes the patient age, IQ, and cognitive test instrument for patients identified in the literature.

### 2.4 Statistical methods

Data were analyzed using the SAS software (Cary, North Carolina, 2009) to perform linear regression and repeated measures linear regression. A *P*‐value less than .05 was considered statistically significant.

## 3 RESULTS

### 3.1 Analysis of cognitive abilities in new patients

**Figure 1:**
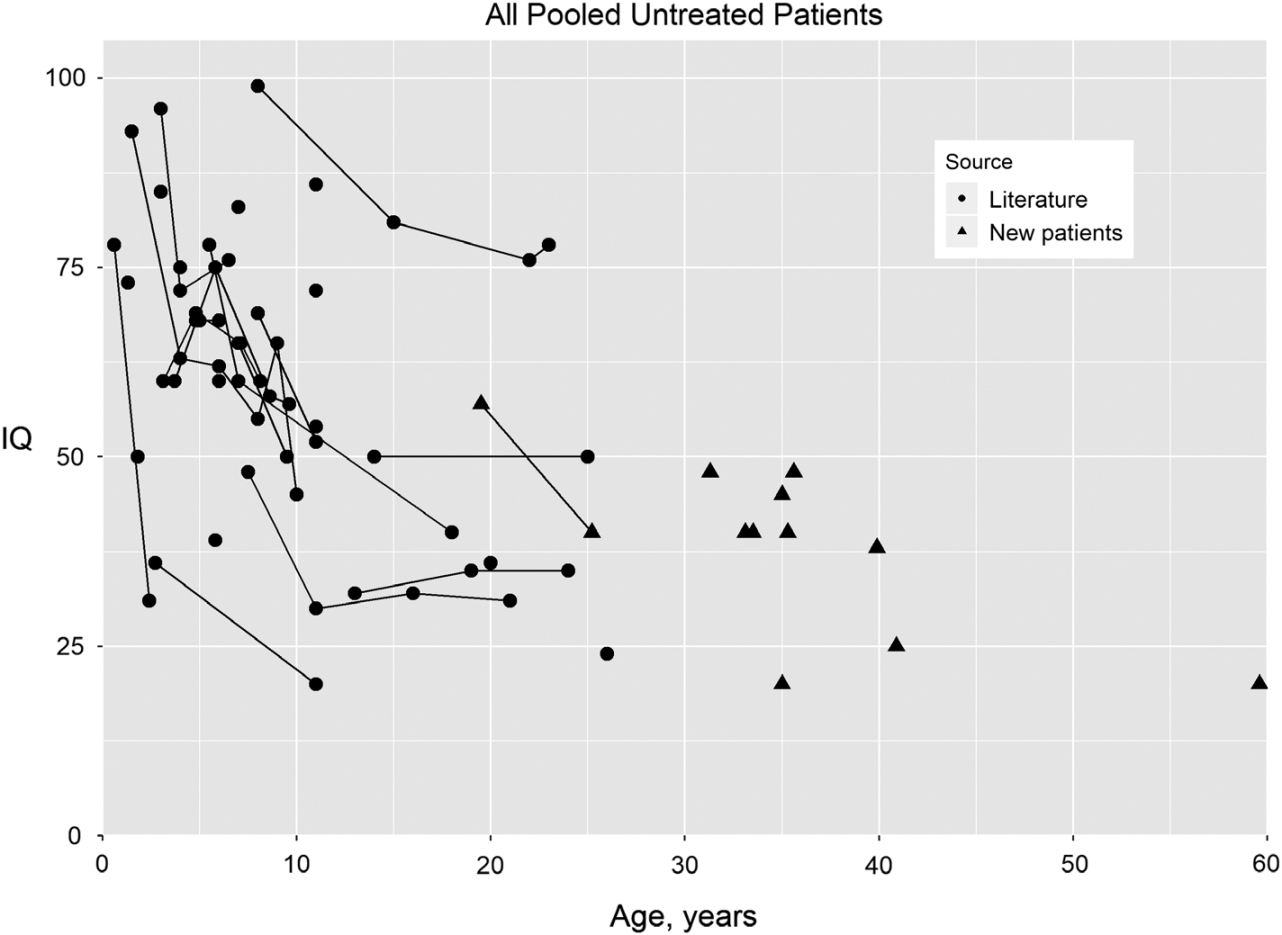
IQ by age for patients with alpha‐mannosidosis. Data points for individuals with multiple evaluations (n = 15) are connected by a line. Patients from the literature (n = 27) are represented with circles and new patients (n = 11) are represented with triangles. See Section 2 for literature citations

Twelve patients with alpha‐mannosidosis (10 males, 2 females) were evaluated in either 2009 or 2012 (Table 1, Table S1). Patient ages at assessment ranged from 8 to 59 years with average age of 34 years. The youngest patient in this cohort was the only patient who had undergone HCT and had a significantly higher IQ standard score of 95. Among the untreated alpha‐mannosidosis patients (ages 25‐59 years), the average IQ was 37 (range 20‐48, Figure 1). Using simple linear regression, a cross‐sectional decline of 0.74 IQ points per year of age was observed across the ages of 25 to 59 years (*P* = .0426) in patients who were not treated with HCT. Removing the oldest patient, potentially an outlier, made the age range 25 to 41 years and did not affect the estimated slope of the regression line but it became nonsignificant (*β* = 0.76, *P* = .3009). Medical records for patient 07 included four IQ measurements over 19 years, documenting significant decline in functioning. IQ was in the “low average range” before age 6, “borderline” at age 6 years, in the mildly intellectually disabled range (IQ = 57) at 19 years, and in the moderately disabled range at age 25 with IQ score of 40. Only the two quantified IQ scores were utilized in data analysis.

**Table 1:** Age, gender, and IQ scores for 12 new patients with alpha‐mannosidosis

Feature	Treated with hematopoietic stem cell transplantation (n = 1)	Untreated (n = 11) Mean (range)
Gender	1 male	9 males, 2 females
Age, years	8	36.8 (25‐59)
IQ standard score	95	36.7 (20–48)

Five of six patients with alpha‐mannosidosis assessed with the K‐BIT‐2 had higher Nonverbal than Verbal IQ scores indicating better visual than verbal problem‐solving skills. This discrepancy may be largely related to the hearing loss commonly associated with alpha‐mannosidosis. The DAYC was administered to three patients (ages 36‐40 years) whose low functioning precluded use of the K‐BIT‐2. The weakest subtest scores were physical and communication domains, with average age‐equivalent scores of 9.7 and 22 months, respectively. These individuals required wheelchairs, had moderate to severe hearing loss, and had limited speech. The PPVT‐4 was administered to two patients who were nonverbal. Both patients scored 20, the lowest standard score possible on this assessment.

### 3.2 Pooled analysis of IQ change with age in new and literature‐based untreated patients

Twenty‐seven untreated patients with measured IQ derived from multiple instruments were identified in the literature (Figure 1). The literature patients were substantially younger (median age 8 years, range of 0.6‐26 years) than the new patients in our cohort (range 25‐59 years). Serial measures were available for 14 patients from the literature and 1 patient from our cohort (range of 2‐6 IQ measures per patient). The longitudinal data for these patients show greater declines in the first decade of life with smaller decline in the second decade. A pooled analysis of our cohort and all untreated literature patients (n = 38) was then performed. Because IQ appears to decline more quickly in the first two decades of life, the analysis was performed separately for patients under age 20 years and patients 20 years and older. For those under age 20 years, using a repeated measures linear regression model, IQ declines at a rate of 1.89 points per year of age (*P* = .0007, intercept = 76.5). In the group aged 20 years and older, only one individual had a second measure so a standard linear regression model was used to estimate age‐related cognitive decline in a cross‐sectional manner. The decline in IQ with age after age 20 years is 0.56 IQ points per year of age (*P* = .0567, intercept = 64.7).

### 3.3 Pooled analysis of changes in IQ with age in patients treated with HCT

**Figure 2:**
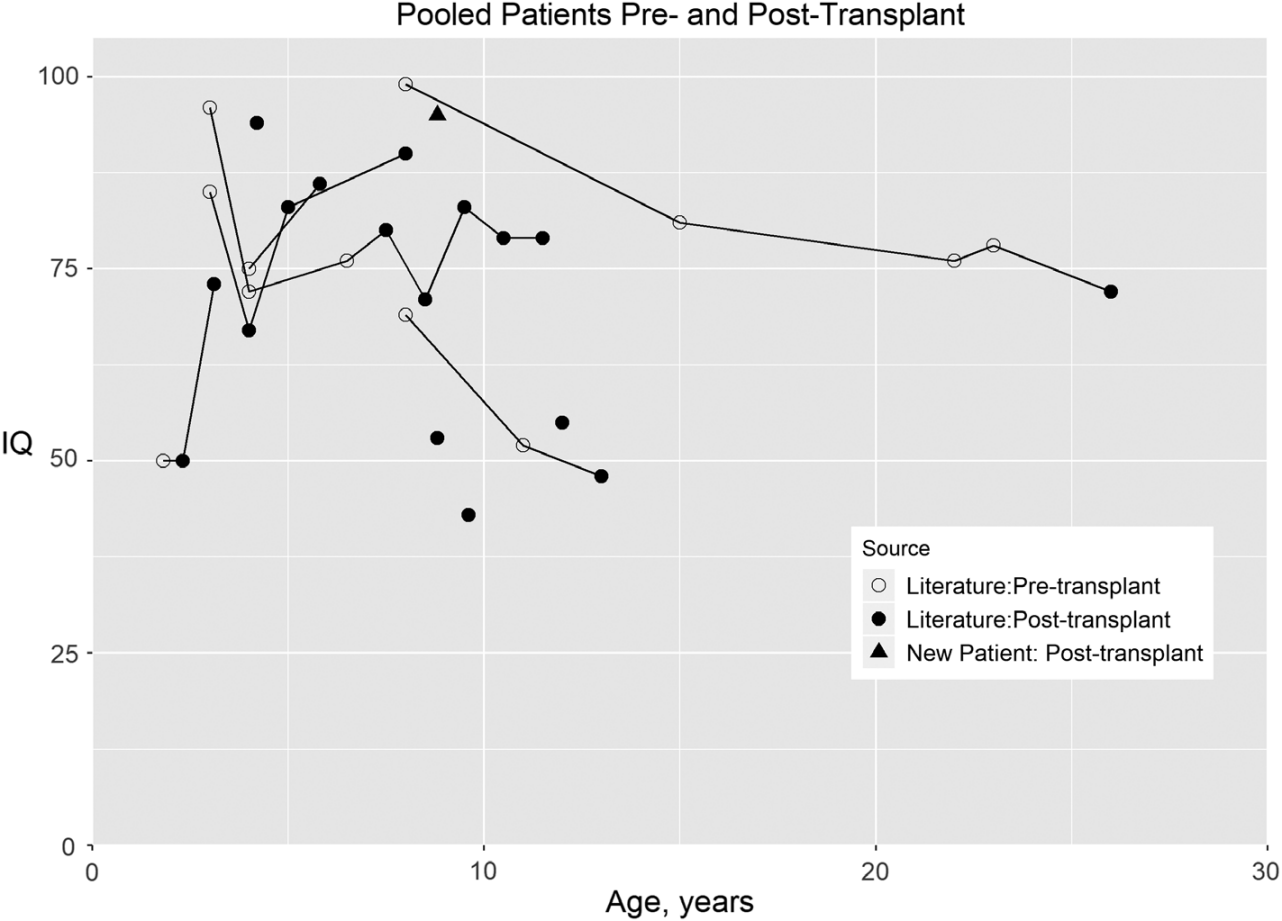
IQ by age for pooled patients pre‐ and post‐HCT. Data obtained from the literature (n = 10) and the present study (n = 1). Data points for individuals with multiple evaluations are connected by a line. Six patients had pre‐transplant IQ measures, which are also included in Figure 1

This study includes 1 new transplanted patient and 10 literature patients who had undergone transplantation (Figure 2, Tables [Link], [Link]). IQ measures were available both pre‐ and post‐transplantation for six of these patients. Transplantation was preceded by substantial declines in IQ. All four patients transplanted before age 10 years showed gains or stabilization in the borderline average to average range of IQ (IQ >70) post‐transplant. The two patients transplanted after age 10 years showed small losses in IQ post‐transplant. Of the five patients without serial measures, two had IQ in the borderline average to average range (IQ > 70) after transplantation. In the transplanted group, eight of 10 (80%) patients ≤20 years of age had an IQ > 50 whereas 15 out of 27 (54%) had an IQ > 50 in the untransplanted group.

## 4 DISCUSSION

Often the diagnosis of a rare disease is accompanied by a medical prognosis, but families are equally interested in a functional prognosis regarding expected levels of school achievement and potential independence throughout the lifespan. We found IQ to vary widely among patients with alpha‐mannosidosis with most, but not all, showing intellectual disability. Untreated patients showed a steep decline in IQ before age 20 years, particularly during the first decade of life, followed by a nonsignificant decline after age 20 in agreement with findings by Autio et al.^15^ Larger longitudinal studies, preferably using a single standardized instrument, are needed to elucidate the pattern of decline.

Borgwardt et al assessed cognitive profiles of 35 patients between 6 and 35 years of age in a cross‐sectional manner using the Leiter International Scale‐Revised.^26^, ^27^ Their cross‐sectional analysis used age‐equivalent or developmental scores rather than standardized IQ scores. Patients show positive cognitive development until age 10 to 12 years and then little change. Still, affected individuals fall increasingly further behind their same‐aged normal peers. This divergence from same‐aged unaffected peers is consistent with our analysis, which showed steep declines in IQ (an age‐normed measure) in the first decade of life with tapering of declines in adulthood. Their patients had a similar range of IQ scores as our cohort with a mean IQ of 42 and a range from 20 to 81, with most patients being in the moderate level of intellectual disability. Borgwardt et al used age‐equivalent and raw developmental scores which have the advantage of describing absolute changes in cognitive development (positive, arrested, or regression) rather than performance relative to same‐aged normal peers.^26^ However, three advantages of using standardized IQ measures as presented in the cohort here are the scores are age‐normed for comparison to typical peers, standardized IQ scores are more frequently reported in the alpha‐mannosidosis literature, and these measures are stable in adulthood.

### 4.1 Hematopoietic stem cell transplant

In this study, we report on cognitive scores of 11 patients with HCT. IQ appears to stabilize after HCT, however, multiple post‐transplant IQ measures were only available for three patients, highlighting the need for future publications on this topic. Newly diagnosed alpha‐mannosidosis patients are now routinely referred to transplantation centers, but published outcome data are still lacking and HCT carries risk.^5^ Wall et al were the first to report stabilization of neurocognitive function in an alpha‐mannosidosis patient treated with bone marrow transplantation.^6^ Over the last 15 years case reports and small series of patients treated with transplantation have been published.^5^, ^13^, ^18^, ^24^ Conditioning regimens varied from patient to patient as did source of stem cells (cord blood, bone marrow, peripheral blood stem cells) and the types of donors (HLA matched or mismatched, related or unrelated to the patient). Follow‐up measures are needed for patients post‐transplantation to confirm the maintenance or improvement in cognitive function long term.

### 4.2 Strengths and limitations

An inherent limitation of studies of individuals affected with a severe rare disease is that disability level may impact participation in such studies. Because this disorder is uncommon and severely affected patients may have shortened lives or difficulties traveling, large cohorts are difficult to assemble. This concern was addressed by testing a relatively large number of patients (n = 12) and pooling data obtained from the literature to create an even larger study size.

Another limitation in assessing age‐related changes is the reliance on cross‐sectional rather than longitudinal data. Additional longitudinal assessments using a single standardized instrument will allow for better delineation of the trajectory of decline, whether it be linear, logarithmic, or multiphasic. The literature often uses broad qualitative descriptors (eg, impaired + or −, “mild,” “moderate”) which obscure the disease progression. A strength of this study was the use of quantitative, measured IQ scores. In future longitudinal studies, use of age‐equivalent scores may be useful for describing cognitive developmental patterns.

## 5 CONCLUSION

Alpha‐mannosidosis is a complex, progressive disorder. The data presented here provide benchmarks of cognitive functioning in a large cohort of patients with alpha‐mannosidosis. The natural history of untreated disease is rapid decline in cognitive function during the first two decades of life. Early HCT appears to stabilize cognitive functioning but timing, preconditioning regimens, and preferred stem cell source have not been fully defined. Too often, assessments of cognitive functioning abilities are omitted from clinical reports, yet these are as important as radiographs and echocardiograms when considering disease progression and effectiveness of interventions. Future studies of alpha‐mannosidosis should include serial measures of cognitive function to provide useful information to patients, families, and clinicians who care for them.

## CONTRIBUTIONS OF AUTHORS

S.S.C. designed and managed the study, interviewed and examined patients, interpreted results, wrote the article, and serves as guarantor. S.M.S. managed data, performed statistical analysis, and wrote the article. R.S., K.P., G.K., D.S., C.W. performed psychological testing and approved the article. L.H. co‐designed the study, performed psychological testing, managed data, and wrote the article.

## COMPLIANCE AND ETHICAL STANDARDS

Sara S. Cathey, Sara M. Sarasua, Richard Simensen, Katie Pietris, Gordon Kimbrell, David Sillence, Callum Wilson, Lucia Horowitz declare they have no conflict of interest.

## INFORMED CONSENT

Written informed consent was obtained from the patient or his/her legally authorized guardian(s) prior to participation in this study. The study was approved by the Institutional Review Board of the Self Regional Health System.

## ANIMAL RIGHTS

This article does not contain any studies with animal subjects performed by the any of the authors.

## Supporting information


**Table S1** Data for 12 Patients with Alpha Mannosidosis Intellectual Functioning in Alpha Mannosidosis.

**Table S2** Alpha‐Mannosidosis Patients from the Literature Intellectual Functioning in Alpha‐Mannosidosis.
