# Supplementary material for: Intellectual functioning in alpha‐mannosidosis
Source: JIMD Rep. 2019 Sep 21;50(1):44–9. doi: 10.1002/jmd2.12073 (PMC6850974; doi:10.1002/jmd2.12073)
Supplement: Supplementary file 1 — Table S1 Data for 12 Patients with Alpha Mannosidosis Intellectual Functioning in Alpha Mannosidosis. [file JMD2-50-44-s001.pdf]

Supplementa1 Table 1: Data for 12 Patients with Alpha Mannosidosis
Intellectual Functioning in Alpha Mannosidosis

ID	Treated with HCT	Gender	Age at diagnosis	First concerns	Age at IQ test, years	IQ Score	IQ Instrument
Patient01	no	male	29 years	concerns for hearing, developemental delays, and recurrent infections in first 2 years of life	59.6	20	PPVT-4
Patient02	no	female	15 years	12-18 months -recurrent ear infections, devepmental delay, hearing loss, hearing aids 4 years	35	20	PPVT-4
Patient03	no	male	26 months	respiratory infections in first few months, recurrent ear infections, enlarged spleen	33.5	40	KBIT-2
Patient04	no	male	6 months	recurrent infections in infancy, hearing aids by age 4 years	31.3	48	KBIT-2
Patient05	no	male	4 years	Poor feeding in infancy, recurrent infections	35.3	40	KBIT-2
Patient06	no	male	2.5 years	unusual appearance of chest at birth, hearing loss and speech regression at 18 months	33.1	40	KBIT-2
Patient07	no	female	4 years	18 months - hearing impairment, delays, kyphosis	19.5	57	Wechsler Adult Intelligence Scale III - from chart review
					25.2	40	KBIT-2
Patient08	no	male	15 years	12-18 months -recurrent ear infections, developmental delay, hearing loss, hearing aids 4 years	35	45	KBIT-2
Patient09	no	male	3 years	by 18 months, hearing loss, delays, recurrent infections; spoke-like cataracts at 3.5 years	35.6	48	DAYC
Patient10	no	male	6 years	12 months delays and developemental regression	40.9	25	DAYC
Patient11	no	male	5 years	developmental delays	39.9	38	DAYC
Patient12	yes	male	21 months	hearing 9 months, kyphosis 10 months, hearing aids 16m, speech delay; unrelated cord blood transplant at 2.5 years.	8.8	95	KBIT-2

IQ Test abbreviations
DAYC, Developmental Assessment of Young Children
KBIT-2, Kaufman Brief Intelligence Test-3
PPVT-4, Peabody Picture Vocabulary Test-4
