# Supplementary material for: Intellectual functioning in alpha‐mannosidosis
Source: JIMD Rep. 2019 Sep 21;50(1):44–9. doi: 10.1002/jmd2.12073 (PMC6850974; doi:10.1002/jmd2.12073)
Supplement: Supplementary file 2 — Table S2 Alpha‐Mannosidosis Patients from the Literature Intellectual Functioning in Alpha‐Mannosidosis. [file JMD2-50-44-s002.pdf]

Supplemental Table 2: Alpha Mannosidosis Patients from the Literature
Intellectual Functioning in Alpha Mannosidosis

Source	Patient ID*	Age at IQ test	IQ	Treated with HCT	IQ instrument	Year of Publication	Notes
Literature	Albert_1	4.2	94	yes	SON (Snijders-Oomen Nonverbal intelligence test)	2003	also published as Mynarek patient 7
Literature	Ara_2	20	36	no	WAIS (Wechsler Adult Intelligence Scale)	1999	
Literature	Autio_1	5.5	78	no	Various instruments, values estimated from graph	1982	
Literature	Autio_1	7	60	no	Various instruments, values estimated from graph	1982	
Literature	Autio_1	18	40	no	Various instruments, values estimated from graph	1982	
Literature	Autio_2	1.5	93	no	Various instruments, values estimated from graph	1982	
Literature	Autio_2	4	63	no	Various instruments, values estimated from graph	1982	
Literature	Autio_2	6	62	no	Various instruments, values estimated from graph	1982	
Literature	Autio_2	8	55	no	Various instruments, values estimated from graph	1982	
Literature	Autio_2	9	65	no	Various instruments, values estimated from graph	1982	
Literature	Autio_2	10	45	no	Various instruments, values estimated from graph	1982	
Literature	Autio_3	2.7	36	no	Various instruments, values estimated from graph	1982	
Literature	Autio_3	11	20	no	Various instruments, values estimated from graph	1982	
Literature	Autio_4	13	32	no	Various instruments, values estimated from graph	1982	
Literature	Autio_4	19	35	no	Various instruments, values estimated from graph	1982	
Literature	Autio_4	24	35	no	Various instruments, values estimated from graph	1982	
Literature	Autio_5	7.5	48	no	Various instruments, values estimated from graph	1982	
Literature	Autio_5	11	30	no	Various instruments, values estimated from graph	1982	
Literature	Autio_5	16	32	no	Various instruments, values estimated from graph	1982	
Literature	Autio_5	21	31	no	Various instruments, values estimated from graph	1982	
Literature	Autio_6	14	50	no	Various instruments, values estimated from graph	1982	
Literature	Autio_6	25	50	no	Various instruments, values estimated from graph	1982	
Literature	Autio_7	7	65	no	Various instruments, values estimated from graph	1982	
Literature	Autio_7	9.5	50	no	Various instruments, values estimated from graph	1982	
Literature	Autio_8	5	68	no	Various instruments, values estimated from graph	1982	
Literature	Aylsworth_KA	0.6	78	no	Bayley Scales MDI (Mental Development Index)	1976	
Literature	Aylsworth_KA	2.4	31	no	Bayley Scales MDI (Mental Development Index)	1976	
Literature	DeFriend	11	72	no	Not specified	2000	
Literature	Grewal_1	3	96	no	Not specified	2004	
Literature	Grewal_1	4	72	no	Not specified	2004	
Literature	Grewal_1	6.5	76	no	Not specified	2004	
Literature	Grewal_1	7.5	80	yes	Not specified	2004	
Literature	Grewal_1	8.5	71	yes	Not specified	2004	
Literature	Grewal_1	9.5	83	yes	Not specified	2004	
Literature	Grewal_1	10.5	79	yes	Not specified	2004	
Literature	Grewal_1	11.5	79	yes	Not specified	2004	
Literature	Grewal_2	3	85	no	Not specified	2004	also published as Mynarek patient 2
Literature	Grewal_2	4	67	yes	Not specified	2004	
Literature	Grewal_2	5	83	yes	Not specified	2004	

Supplemental Table 2: Alpha Mannosidosis Patients from the Literature
Intellectual Functioning in Alpha Mannosidosis

Source	Patient ID*	Age at IQ test	IQ	Treated with HCT	IQ instrument	Year of Publication	Notes
Literature	Grewal_2	8	90	yes	Not specified	2004	
Literature	Grewal_3	8	99	no	Not specified	2004	
Literature	Grewal_3	15	81	no	Not specified	2004	
Literature	Grewal_3	22	76	no	Not specified	2004	
Literature	Grewal_3	23	78	no	Not specified	2004	
Literature	Grewal_3	26	72	yes	Not specified	2004	
Literature	Grewal_4	4	75	no	Not specified	2004	also published as Mynarek patient 10
Literature	Grewal_4	5.8	86	yes	Not specified	2004	
Literature	Kistler	26	24	no	Stanford-Binet	1977	
Literature	Mynarek_3	7	83	no	WISC-R (Wechsler Intelligence Scale for Children)	2012	
Literature	Mynarek_4	12	55	yes	DAS-II (Differential Ability Scales, 2nd edition)	2012	
Literature	Mynarek_6	1.3	73	no	DQ, CAT/CLAMS (Developmental Quotient, Cognitive A	2012	
Literature	Mynarek_9	9.6	43	yes	WISC-III (Wechsler Intelligence Scale for Children)	2012	
Literature	Niemann	6	60	no	Not specified	1996	
Literature	Noll_1	3.7	60	no	Stanford-Binet	1989	
Literature	Noll_1	5.8	75	no	Stanford-Binet	1989	
Literature	Noll_1	8.6	58	no	Stanford-Binet	1989	
Literature	Noll_1	9.6	57	no	Stanford-Binet	1989	
Literature	Noll_2	3.1	60	no	Stanford-Binet	1989	
Literature	Noll_2	4.8	69	no	Stanford-Binet	1989	
Literature	Noll_2	7.1	65	no	Stanford-Binet	1989	
Literature	Noll_2	8.1	60	no	Stanford-Binet	1989	
Literature	Noll_3	4.8	68	no	Stanford-Binet	1989	
Literature	Noll_3	6	68	no	Stanford-Binet	1989	
Literature	Segoloni	11	54	no	Wish method	1996	
Literature	Urushihara	11	86	no	Not specified	2004	
Literature	Wall_1	1.8	50	no	Bailey Scales of Infant Development	1998	
Literature	Wall_1	2.3	50	yes	Bailey Scales of Infant Development	1998	
Literature	Wall_1	3.1	73	yes	Stanford-Binet	1998	
Literature	Yesilipek_1	8	69	no	Stanford-Binet	2012	
Literature	Yesilipek_1	11	52	no	Stanford-Binet	2012	
Literature	Yesilipek_1	13	48	yes	Stanford-Binet	2012	
Literature	Yesilipek_2	8.8	53	yes	Stanford-Binet	2012	
Literature	Yunis_1	5.8	39	no	Not specified	1976	

*Patient ID is the first author of the original publication followed by patient identifier for the data obtained from the literature
